# Radiofrequency ablation for thyroid nodules in Ecuador: a cross-sectional study

**DOI:** 10.1186/s13044-023-00188-y

**Published:** 2024-01-03

**Authors:** Cristhian Garcia, Paola Solis-Pazmino, Eddy P. Lincango, Andrea S. Cho-Tana, Luis Figueroa, Oscar J. Ponce, Juan P. Brito, Erivelto Volpi

**Affiliations:** 1 Instituto de Tiroides y Enfermedades de Cabeza y Cuello (ITECC), Quito, Ecuador; 2 The Surgery Group of Los Angeles, 8635 W 3rd St #880, Los Angeles, CA 90048 USA; 3 Knowledge and Evaluation Research Unit, Mayo Clinic, Rochester, MN 55905 USA; 4 Division of Endocrinology, Diabetes, Metabolism, and Nutrition, Mayo Clinic, Rochester, MN 55905 USA; 5 Oncology Center, Oswaldo Cruz German Hospital, Sao Paulo, Brazil; 6 Duque de Caxias, Porto Alegre 1667 Brazil

**Keywords:** Benign thyroid nodule, Microcarcinoma, Radiofrequency

## Abstract

**Objectives:**

To describe the demographic characteristics and clinical outcomes following the first cohort of patients with Bening Thyroid Nodule (BTN) and (Papillary Thyroid Microcarcinoma) (PTMC) treated with Radiofrequency Ablation (RFA)in Ecuador.

**Methods:**

Single-center, cross-sectional study. We included adults undergoing RFA for BTN and PTMC between July 2019 and May 2022. Descriptive statistics and the Wilcoxon signed-rank test were used to compare some pre- and post-intervention outcomes.

**Results:**

We included 44 patients with 36 BTNs and eight PTMCs. The median age was 45.80 years (IQR 16–79 years), and most patients had normal thyroid function (72.72%). The median follow-up time was 7.80 months (IQR1.0-34.0). Nodules were primarily solid (43.21%) or predominantly solid (56.81%). The pre-RFA median volume in the benign lesions group was 10.30 ml (IQR 1.86–18.97). After ablation, the 1-month, 3-month, 6-month, and 12-month median volumes were 6.90 (IQR 0.48–10.15; p < 0.01) mL, 5.72 (IQR 0.77–7.25; p = 0.045); 0.98 (IQR 0.25–3.64; p < 0.01), and 0.11 (IQR 0.07–11.26; p = 0.026), respectively. The volume rate reduction was 47.20%, 72.20%, 74.00%, and 96.20% at 1, 3, 6, and 12-month follow-ups, respectively. The pre-RFA median volume in the PTMC group was 0.25 ml (IQR 0.19–0.48). After ablation, the 1-month, 3-month, and 6-month mean volumes were 0.19 (range 0.12–0.31; p = 0.120) mL, 0.10 (IQR 0.05–0.15; p = 0.13), and 0.01 (IQR 0.005–0.04; p = 0.364), respectively.

**Conclusions:**

In this first report from Ecuador, we found that RFA may be a feasible alternative for treating benign and malignant thyroid nodules in the short term. Long-term data are needed to evaluate oncologic outcomes in PTMC patients.

## Key Points

RFA may be a feasible alternative for the short-term treatment of benign and malignant thyroid nodules.

Thyroid nodules were ablated in a single session, having a significant volume reduction rate and improved cosmetic and symptom scores after RFA.

The study highlights the need for long-term follow-up to assess the oncologic outcomes in PTMC patients who undergo RFA.

## Introduction

The incidence of palpable thyroid nodules (TN) ranges from 3 to 7% worldwide [1]. While most nodules do not lead to mortality, some can cause compressive symptoms or cosmetic concerns because of their size and location. On the other hand, just about 5% of all TNs are malignant and need surgery; a third of these cases are papillary thyroid microcarcinoma (PTMC) (1 cm or less in size). PTMCs are primarily indolent, with an excellent prognosis. Traditionally, surgery treats symptomatic TN and PTMC^2^; however, new ablative therapies could safely treat them without removing the gland.

Radiofrequency ablation (RFA) has emerged as an effective and safe treatment strategy for solid TN [2] and PTMC. In 2008, a Korean group published one of the first papers on a benign thyroid nodule cohort treated with RFA, where there was an approximately 85% volume reduction after six months [3]. Other experiences with solid nodules have shown similar or superior results [4]. Although RFA has not been established as the gold standard for low-risk PTMC, evidence suggests that RFA is a feasible treatment option for low-risk PTMC in patients who refuse surgery or are ineligible [5]. To our knowledge, five countries (Ecuador, Brazil, Colombia, Argentina, and Peru) use RFA in TN treatment in Latin America.

RFA allows the preservation of the thyroid gland and preserves thyroid function with minimal risk of procedural.

Complications [6–8]. Considering this, an international panel comprising surgeons, endocrinologists, and radiologists with expertise in RFA recommended RFA for benign and malignant thyroid nodules [9]. Our study aims to describe the demographic characteristics and clinical outcomes following the first cohort of patients with TN who underwent ablative radiofrequency in Ecuador.

## Methods

The Universidad San Francisco de Quito approved the institutional review board (IRB). This study followed the STROBE guidelines for observational studies [10]. All the patients signed the informed consent before the RFA procedure.

### Setting and participants

This single-center, cross-sectional study was conducted at the Instituto de la tiroides y Enfermedades de Cabeza y Cuello (ITECC), a private reference center for patients with thyroid nodules and thyroid cancer in Quito, Ecuador. From July 2019 to May 2022, we included a convenience sample of 44 patients who underwent RFA for benign non-functional TN and PTMC performed by a head and neck surgeon (C.G.).

Thyroid nodules were considered benign if (i) two separate fine-needle aspirations (FNA) cytology biopsies reported benign features or (ii) one FNA reported benign features with ultrasonographic (US) characteristics highly specific for benignity (e.g., spongiform). Similarly, unifocal PTMCs were confirmed by fine-needle aspiration cytology biopsies (Bethesda 5 and 6). To be included, patients with PTMC needed to fulfill the following criteria: (i) a maximum diameter no larger than 10 mm; (ii) absence of capsular infiltration and extrathyroidal invasion in the US; and (iii) no cervical lymph node metastasis in the US. Patients who received thyroid treatments within the year before the study period, those without follow-up information, and patients or caregivers who refused to participate were excluded from the study.

### Pre-ablation assessment

Before treatment, patients had complete blood count, thyroid function tests, coagulation tests, and imaging evaluation. The US collected features including size, location, margin, shape, echogenicity, calcification, and vascularity. The nodule volume was calculated according to the American Thyroid Association (ATA) calculator [11] (formula: V = πabc/6 - where *V* is the volume, *a* is the largest diameter in the US, and *b* and *c* are the other two perpendicular diameters).

Nodules were categorized based on the ratio of cystic to solid portion into solid (no apparent cystic content), predominantly solid (cystic amount ≤ 50% of nodule), predominantly cystic (cystic part > 50% of nodule), and cystic (no apparent solid content) [12]. Also, we defined mixed TN when the solid component was less than 70% and greater than 30%.

We employed a classification system, described in a previous consensus statement, to assess the severity of symptoms and the impact on cosmetic appearance [13]. Patients were asked to rate their pressure symptoms on a 10-cm visual analog scale, with a range of 0 to 10 cm, at the initiation of the study (same day of RFA) and during subsequent visits (1, 3, 6, and 12 months). The cosmetic score was evaluated using a 4-point scale, which included the following categories: (1) absence of a palpable mass; (2) palpable mass with no cosmetic impact; (3) cosmetic impact only during swallowing; and (4) easily visible mass.

### Ablation technique

RFA was performed by a head and neck surgeon using local anesthesia (2% lidocaine without epinephrine). The technical feasibility assessment focused on an easily accessible microprocessor, Mygen M-3004, with an internally cooled electrode (18-gauge, 5 mm, or 7 mm active tip) according to the nodule’s size and nature (benign or malignant). Ultrasound guidance linear transducer L6-12 RS / GE with a trans-isthmic approach and moving shot technique with 20 to 40 W of power was applied [14]. Ablation termination was determined when all visual fields of the nodule had changed to transient hyperechoic zones. For those TN felt to be hugely fulfilled of cystic liquid, aspiration with a G18 syringe was performed before RFA. This equipment is available in our institution for all our patients. Moreover, our patients stay in our clinic for a couple of hours post-RFA and then go home the same day.

### Follow-up evaluation

Patients were followed up at 1, 3, 6, and 12 months after index RFA. During these appointments, we evaluated the cosmetic and symptomatic scores. Also, US and laboratory tests were required at each follow-up visit. The (VRR), which was applied to assess the extent of nodule volume reduction, was determined using the ATA calculator. Effective treatment was defined as a volume reduction > 50% of the initial nodule volume on a follow-up US examination.

### Analysis and statistics

Statistical analysis was performed by using the R program. The normality of distribution was assessed visually and using the Kolmogorov-Smirnov test. For continuous variables, we calculated the median and interquartile range. Categorical variables were presented by frequency (percentage). We used a paired t-test to assess the pre-and post-RFA VRR changes, laboratory values of stimulating thyroid hormone (TSH), and free thyroxine (fT4). The Wilcoxon signed-rank test compares the pre-and post-RFA cosmetic score changes. We used pairwise deletion to address missing data during the statistical analysis.

## Results

### Demographic characteristics

Table 1 shows the clinical characteristics of the patients who underwent RFA. A total of 44 patients (37 female, 84.1%) were enrolled in this study. The median age was 45.8 years (IQR 16–79), and most patients had normal thyroid function (72.7%). Thirty-six patients had BTN (30 single nodules, six multi-nodular), and eight had PTMC. All the patients required one session of RFA, and most BTNs were located on the right side (65.9%). Nodules were primarily solid (43.2%) or predominantly solid (56.8%). More than half (61.4%) were symptomatic TN. The median follow-up time was 7.8 months (IQR 1–34). We had missing data during this time.

**Table 1:** Baseline Characteristics

Variables	Total (n = 44)	Benign (n = 36)	Microcarcinoma (n = 8)
**Sex**			
Female	37 (84.0%)	31 (84.0%)	6 (16.0%)
Male	7 (16.0%)	5 (71.4%)	2 (28.6%)
**Age at diagnosis**			
Mean (standard deviation)	45.77 (11.9)	47.91 (16.89)	43.63 (7.06)
< 18 years old	2 (4.5%)	2 (100%)	0 (0%)
18–64 years old	35 (79%)	28 (80%)	7 (20%)
≥ 65 years old	7 (16.5%)	6 (86%)	1 (14%)
**Residence**			
Coast	6 (13.6%)	6 (100%)	0 (0%)
Highland	35 (79%)	27 (77%)	8 (13%)
Other countries	3 (7.4%)	3 (100%)	0 (0%)
**Employment**			
Domestic chores	8 (18.2%)	6 (75%)	2 (25%)
Student	4 (9.1%)	4 (100%)	0 (0%)
Labor	32 (72.7%)	26 (81.3%)	6 (18.7%)
**Education level**			
None	0	0	0
Elementary School	3 (6.8%)	1 (33%)	2 (67%)
High school	12 (27.3%)	12 (100%)	0 (0%)
University	29 (65.9%)	23 (79%)	6 (21%)
**Family history of thyroid cancer**			
Yes	7 (16%)	5 (71%)	2 (29%)
No	37 (84%)	31 (84%)	6 (16%)
**BMI**			
Normal	14 (31.8%)	8 (57%)	6 (43%)
Overweight	28 (63.6%)	27 (96%)	1 (4%)
Obesity	2 (4.6%)	1 (50%)	1 (50%)
**Cigarette Smoking**			
Yes	1	1 (100%)	0
No	43	35 (81.4%)	8 (19.6%)
**Thyroid function**			
Euthyroid	32 (72.7%)	25 (78%)	7 (12%)
Hypothyroidism	12 (27.3%)	11 (92%)	1 (8%)
Hyperthyroidism	0	0	0
**Nodule composition**			
Solid	19 (43.2%)	11 (58%)	8 (42%)
Predominantly solid	25 (56.8%)	25 (100%)	0
Mixed	0	0	0
**Laterality**			
Right lobe	29 (65.9%)	26 (89%)	3 (11%)
Left lobe	12 (27.3%)	9 (75%)	3 (25%)
Isthmus	3 (6.8%)	1 (33.3%)	2 (66.7)
**Methods of detection**			
Symptomatic nodule	27 (61.4%)	27 (100%)	0
Asymptomatic patient, screening detection, palpable nodule	11 (25%)	5 (45.5%)	6 (54.5%)
Incidental, asymptomatic patient	6 (13.6%)	4 (66.7%)	2 (33.3%)
Nodule detected in the image for other causes	0	0	0

### Volume reduction ratio (VRR)

#### Benign thyroid nodule (BTN)

**Fig. 1:**
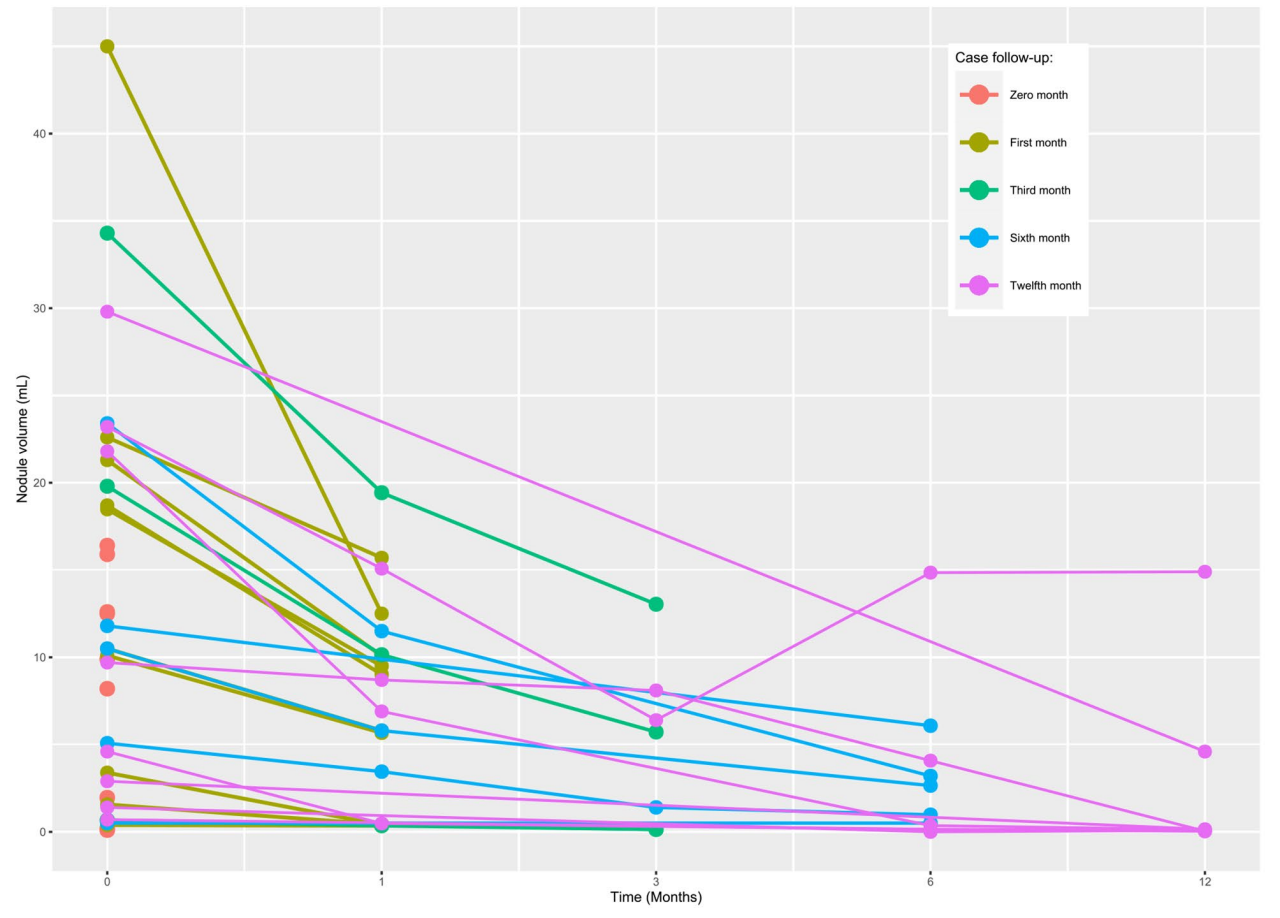
Individual analysis of each benign nodule’s volume (mL) changes over time. Each line represents a separate nodule; time 0 month indicates the day of the RFA procedure, with the length of the line representing the length of the follow-up period. Each circle represents a point in time where the volume of the nodule was measured by ultrasonography, and the points are connected by lines to give an approximate volume reduction rate. After ablation, the 1-month, 3-month, 6-month, and 12-month median volumes were 6.9 (IQR 0.48–10.15; p > 0.0001) mL, 5.72 (IQR 0.77–7.25; p = 0.045) mL; 0.98 (IQR 0.25–3.64; p = 0.007) mL, and 0.11 (IQR 0.07–11.26; p = 0.026) mL, respectively

**Fig. 2:**
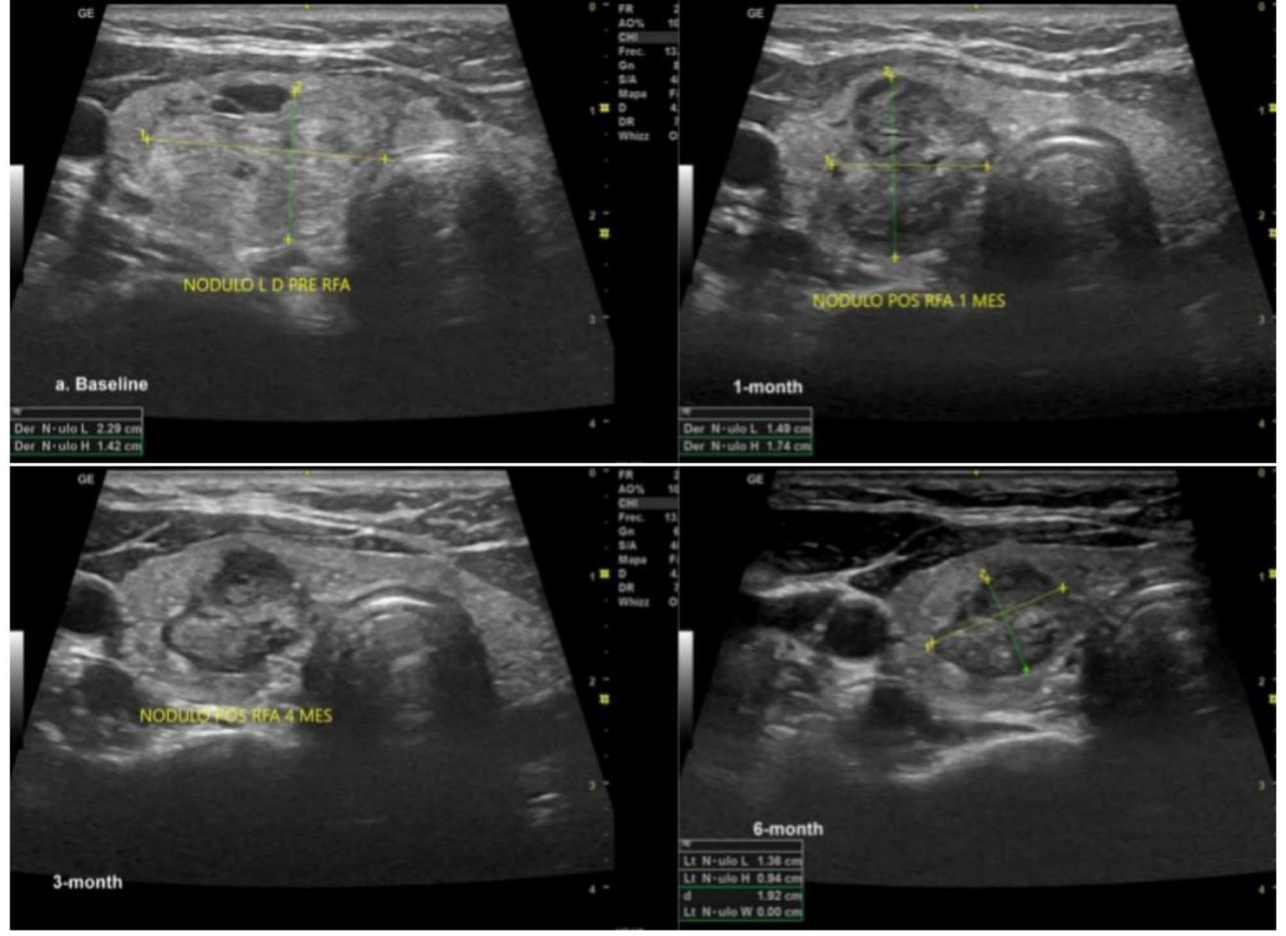
Benign thyroid nodules pre- and post-RFA (1, 3, and 6 months), see the changes in volume and the ultrasound aspect of the nodule due to coagulative necrosis caused by the heat

Before RFA, the overall median baseline volume of the BTNs was 10.30 (IQR 1.86–18.97) mL. After ablation, the 1-month, 3-month, 6-month, and 12-month median volumes were 6.90 (IQR 0.48–10.15; p < 0.001) mL, 5.72 (IQR 0.77–7.25; p = 0.045) mL; 0.98 (IQR 0.25–3.64; p = 0.007) mL, and 0.11 (IQR 0.07–11.26; p = 0.026) mL, respectively. Moreover, the results showed that the overall nodular volume reduced significantly after RFA treatment over time (p < 0.001). The VRR was 47.20%, 72.20%, 74.0%, and 96.20% at 1, 3, 6, and 12-month follow-ups, respectively (Table 2). Figure 1 shows the change in volume of each patient’s nodule volume over time. Each line represents a separate patient. Figure 2 illustrates a TN pre- and post-RFA.

**Table 2:** Ultrasound features and cosmetic and symptom scores in patients before and after the procedures

	Radiofrequency ablation benign nodule (n = 36)						Radiofrequency Microcarcinoma (n = 8)				
	Baseline	1 m	3 m	6 m	12 m	*p*-value	Baseline	1 m	3 m	6 m	12 m
Longest tumor diameter (mm)	36 (21–43) n = 36	31 (12–35) n = 22 p = < 0.001	33 (11-38.5) n = 7 p = 0.0042	20 (11–21) n = 9 p = 0.0025	10 (7–17) n = 8 p = 0.0018	< 0.05	8.5 (7–10) n = 8	9 (7.5–10) n = 4 p = 0.2319	8 (5.5–8.5) n = 3 p = 0.7157	3 (1.5-5) n = 3 p = 0.09418	-
Tumor volume (ml)	10.3 (1.86–18.97) n = 36	6.9 (0.48–10.15) n = 21 p = 0.00021	5.72 (0.77–7.25) n = 7 p = 0.045	0.98 (0.25–3.64) n = 11 p = 0.0077	0.11 (0.07–11.26) n = 8 p = 0.0264	< 0.05	0.25 (0.19–0.48) n = 8	0.19 (0.12–0.31) n = 4 p = 0.1168	0.10 (0.05–0.15) n = 3 p = 0.1306	0.01 (0.005–0.04) n = 3 p = 0.3642	-
Volume reduction ratio (%)	-	47.25 (36.38–64.17) n = 22	72.20 (62-73.20) n = 5 p = 0.019	74 (49–82) n = 9 p = 0.032	96.20 (85.4–97.80) n = 7 p = 0.076	< 0.05	-	42 (64-81.35) n = 3	52.5 (29.59–75.42) n = 2	94.85 (92.28–97.42) n = 2	-
TSH	1.71 (1.4–2.45) n = 32	1.37 (0.81–2.12) n = 8 p = 0.97	2.6 (1.69–3.46) n = 6 p = 0.578	1.97 (1.31–2.52) n = 6 p = 0.33	2.32 (1.88–2.66) n = 6 p = 0.24	> 0.05	1.43 (041-2.72) n = 6	2.27 (2.14–2.75) n = 4 p = 0.1614	2.14 (2.09–2.17) n = 3 p = 0.092	1.30 (1.3) n = 2	-
Cosmetic score (1–4)	2.00 (1–2) n = 36	1.00 (1–1) n = 16	1.00 (1–1) n = 10	1.00 (1–1) n = 9	1.00 (1–1) n = 4	< 0.001	1 (1–2) n = 8	1 (1) n = 6 p = 0.3632	1 (1) n = 3 p = 0.4226	1 (1) n = 2 p = NA	1 (1) n = 2 p = NA
Symptom score (0–10)	2.5 (1.75–4.25) n = 36	1.00 (1–1) n = 16	1.00 (1–1) n = 12	1.00 (1–1) n = 7	0 (0–0) n = 4	< 0.001	0 (0–1) n = 8	0 (0) n = 5 p = 0.3739	0 (0) n = 3 p = NA	0 (0) n = 2 p = NA	0 (0) n = 2 p = NA

Numbers are calculated with median and interquartile ranges

The time, power, and energy delivered by the procedure were 5.17 (SD 8.26 min), 2085.90 (SD 6476.73 W), (10/22), and 13,948 (SD 10936.10 J), respectively.

#### Papillary thyroid microcarcinoma

**Fig. 3:**
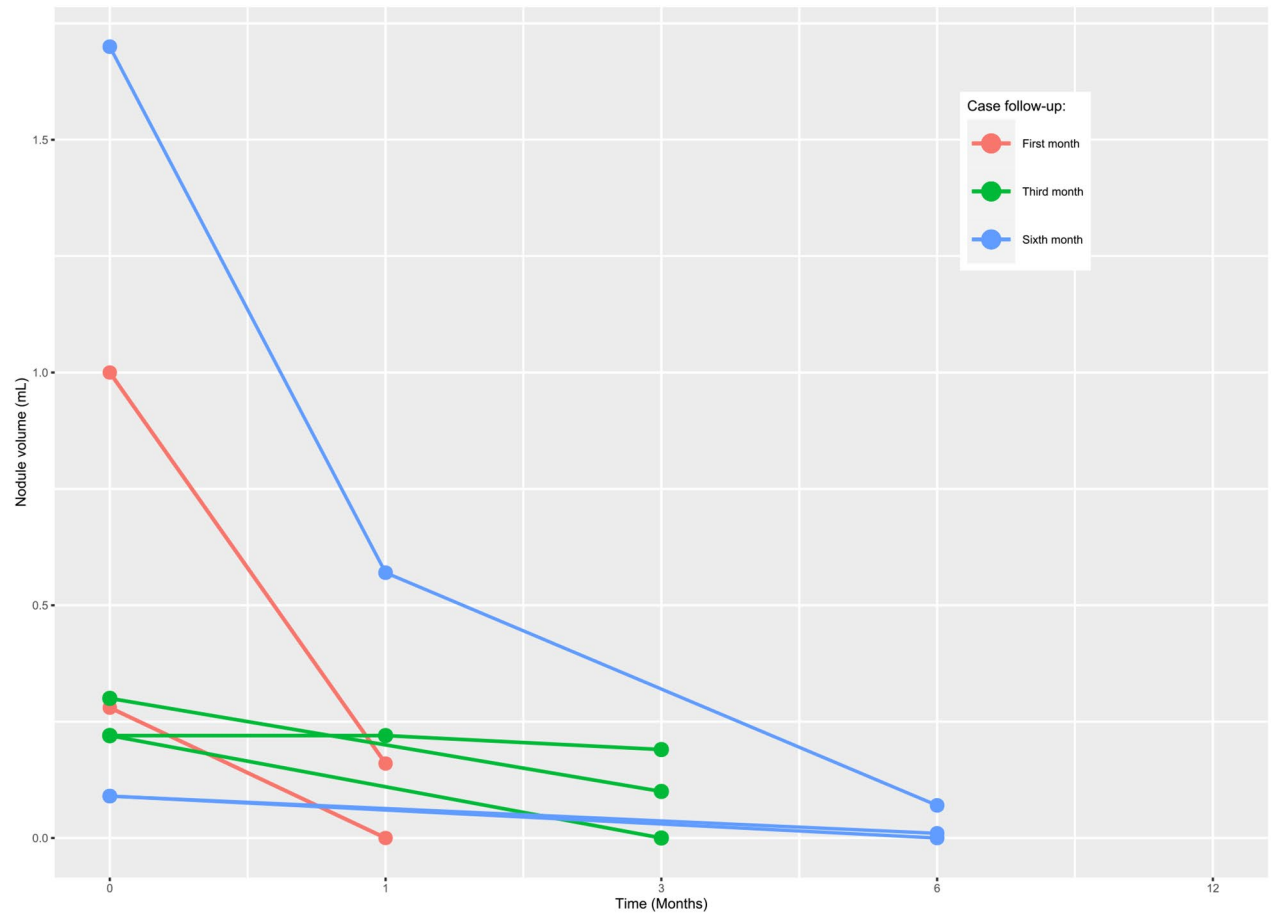
Individual analysis of each malignant nodule’s volume (mL) changes over time. Each line represents a separate nodule; time 0 month indicates the day of the RFA procedure, with the length of the line representing the length of the follow-up period. Each circle represents a point in time where the volume of the nodule was measured by ultrasonography, and the points are connected by lines to give an approximate volume reduction rate. After ablation, the 1-month, 3-month, and 6-month mean volumes were 0.19 (IQR 0.12–0.31; p = 0.190) mL, 0.10 (IQR 0.05–0.15; p = 0.130), and 0.01 (IQR 0.005–0.04; p = 0.360), respectively

**Fig. 4:**
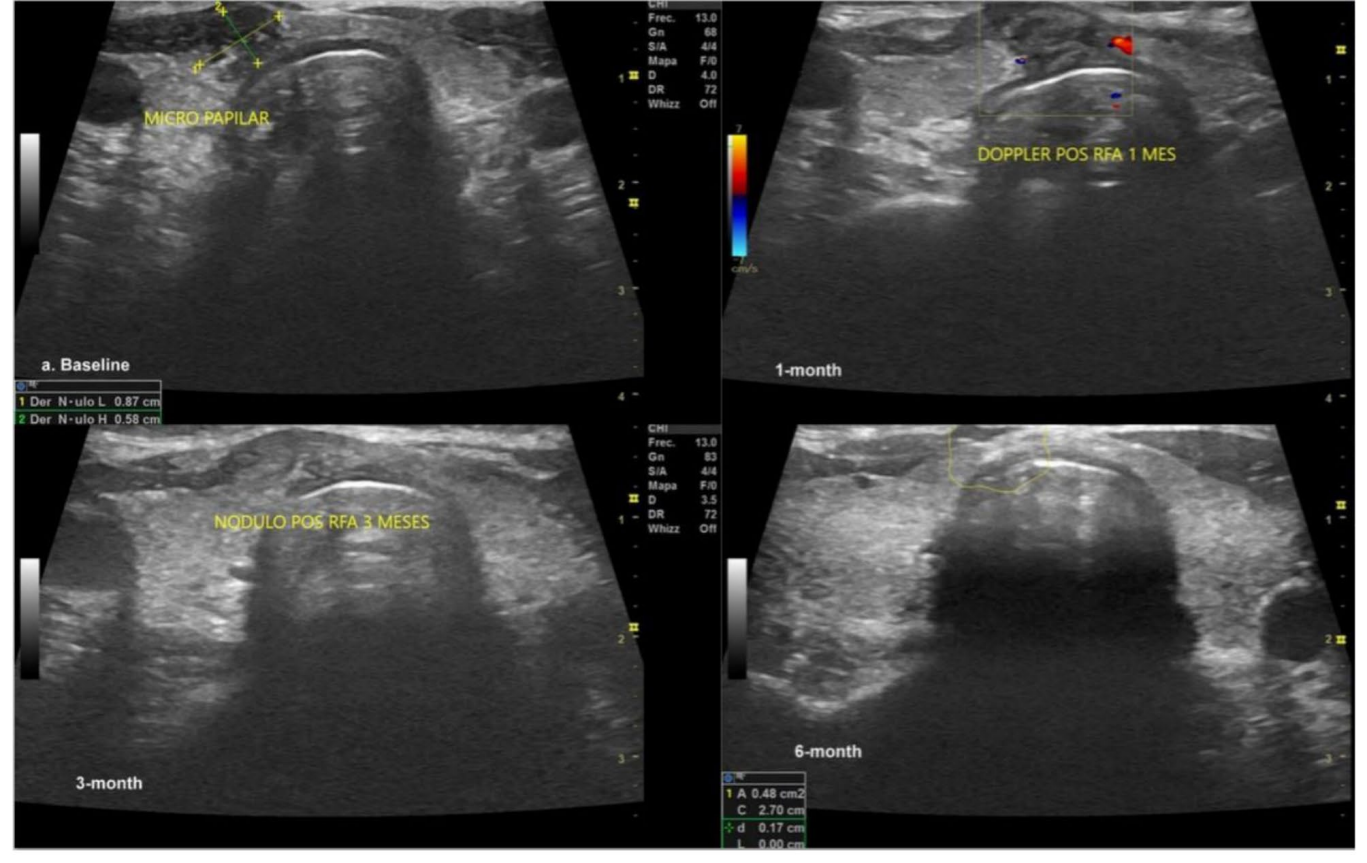
Papillary thyroid microcarcinoma pre- and post-RFA (1, 3, and 6 months). Note that the nodule’s vanishing, making it hard to identify it by ultrasound

VRR changes are shown in Table 2. Before RFA, the overall median baseline volume of the PTMCs was 0.25 (IQR 0.19–0.48) mL. After ablation, the 1-month, 3-month, and 6-month mean volumes were 0.19 (IQR 0.12–0.31; p = 0.190) mL, 0.10 (IQR 0.05–0.15; p = 0.130), and 0.01 (IQR 0.005–0.04; p = 0.360), respectively. Moreover, the results demonstrated that the overall nodular volume reduced after RFA treatment over time VRR was 42.0%, 52.5%, and 94.8% at 1, 3, and 6-month follow-ups, respectively. Figure 3 shows the change in volume of each patient’s nodule volume over time. Each line represents a separate patient. Figure 4 shows an example of a TN pre- and post-RFA.

The time, power, and energy delivered of the procedure were 0.11 (SD 0.00), 37.50 (SD 3.54 W), (10/22) 12,125 (SD 13771.61 J), respectively.

### Cosmetic and symptomatic scores

Table 2 shows the cosmetic and symptomatic scores findings. In the BTN group, the overall cosmetic score improved from 2.0 (IQR 1–2) to 1.0 (IQR 1–1) (p < 0.001) at 3 (n = 10), 6 (n = 9), and 12 months (n = 4). The overall symptomatic score improved from 2.50 (IQR 1.75–4.25) to 0.0 at 3 (n = 12), 6 (n = 7), and 12 months (n = 4).

In the microcarcinoma group, the overall cosmetic and symptomatic scores were low in both the baseline and the follow-up without significant changes.

### Complications

There were only minor complications, including ecchymosis (n = 2, 5.5%) and skin discoloration (n = 3, 8.3%) in patients with BTNs.

## Discussion

This study retrospectively examined radiofrequency ablation (RFA) for Ecuador’s non-functional thyroid nodules and papillary thyroid microcarcinomas. The study found that all TNs were ablated in a single session, having a significant volume reduction rate and improved cosmetic and symptom scores after RFA.

For benign, nonfunctioning thyroid nodules, RFA should be restricted to patients with symptoms or cosmetic issues [15]. The success of RFA treatment is determined objectively with a VRR of at least 50% [2], normal thyroid function studies, and validated instruments (cosmetic questionnaires and symptom scores) [9]. Our study’s VRR at the 12-month follow-up was 88.4%. This is aligned with the findings of a recent meta-analysis. They reported a volume reduction rate at 12 months ranging from 67–75% [4].

The degree of volume reduction achieved with RFA can depend on several factors, including the initial size and characteristics of the nodule, the number of RFA sessions performed, and the technique used during the procedure. For instance, some studies have suggested that a larger starting nodule size may be associated with a lower volume reduction after RFA. However, our study found that patients with larger nodules have more significant volume reduction rates. This is interesting, and we believe the aspiration used before the ablation plays a role. This aspiration is part of the RFA procedure reporting in the Korean guideline [16] and other studies [17].

As with benign thyroid nodules, RFA is becoming part of the decision-making process between patients with small papillary thyroid cancer and their clinicians. Some studies support using RFA in low-risk PTMCs [18–22]. In a recent meta-analysis of 1770 patients who underwent RFA (mean follow-up time of 33.0 months), the pooled complete disappearance rate of PTMCs at the end of follow-up was 79% (95% CI, 65-94%) [5]. In our study, the rate at 1-month follow-up was 42%, whereas at 12-month follow-up was 89.7%. However, we can not make any conclusion at longer follow-ups.

In the same way, some systematic reviews assessing the safety of thermal ablation compared surgery versus RFA and showed that both are safe options for managing benign TN and low-risk PTMCs. However, thermal ablation achieved a lower complication rate [23, 24]. In our study, there were only five minor complications. It demonstrates that RFA offers a potential therapeutic alternative to mitigate some disadvantages of surgical treatment or radioactive iodine ablation.

However, RFA is not appropriate for all thyroid nodules. It is typically recommended for nodules that are causing significant symptoms or that are growing in size. Nodules suspicious of cancer or located in some regions of the thyroid gland may not be suitable for RFA. The minimally invasive techniques, like RFA, impact patient care and help to guide the decision of which treatment most closely aligns with patients’ beliefs and expectations. This may be especially important for improving adherence to patients with contraindications to undergo surgery. Nevertheless, RFA is not appropriate for all types of thyroid nodules. It is recommended for nodules that cause significant symptoms or grow in size. Nodules suspicious of cancer or located in some regions of the thyroid gland may not be suitable for RFA. Therefore, carefully evaluating each case is necessary to determine the most appropriate treatment option.

According to several systematic reviews, both RFA and surgery are safe options for managing benign thyroid nodules (TN) and low-risk papillary thyroid microcarcinomas (PTMCs). However, RFA appears to be associated with a lower complication rate than surgery^29,30^. Our study observed only five minor complications, suggesting radiofrequency ablation (RFA) could be a promising alternative to surgical treatment or radioactive iodine ablation. A Brazilian prospective randomized comparative study found that RFA represented 76% of the cost of partial thyroidectomy, and both had similar complications. However, RFA had a shorter operating time and hospital stay, making it an effective alternative in treating benign thyroid nodules [25].

### Limitation

Our study has some limitations. First, the small sample size did not allow us to draw strong conclusions. Similarly, we could not report the impact of RFA in the long term due to our short follow-up period. Another area for improvement is the need for more data. We assumed that our data were missing at random type. We used pairwise deletion to address missing data during the statistical analysis. It involved calculating the mean of only the available data for each pair of cases rather than deleting the entire observation with missing data. In this method, only the missing values were excluded from the calculation, while the available values were included. Finally, these limitations can be managed with an experimental study design, which helps to decrease the confounding bias in the non-adjusted analysis. Aside from this, our study is a solid contribution to the literature. We documented the feasibility and effectiveness of performing RFA in treating benign and malignant thyroid nodules in our country, the third country in Latin America after Brazil and Colombia. Even with a short follow-up, our results show that the technique is promising and can benefit patients with benign and malignant thyroid nodules when correctly indicated. Therefore, a more extensive series and extended follow-up are necessary to confirm our initial impression.

## Conclusion

In this first report from Ecuador, we found that RFA was a feasible alternative for treating benign and malignant thyroid nodules in the short term. Long-term data are needed to evaluate oncologic outcomes in PTMC patients.

## Declarations

### Clinical relevance

The study demonstrates the feasibility of RFA as an effective treatment option for BTN and PTMC. The findings help clinicians identify candidates for RFA and suggest that it can reduce the volume of both benign and malignant nodules.

## Abbreviations

RFA: Radiofrequency ablation
BTN: Benign thyroid nodules
PTMC: Papillary thyroid microcarcinomas
ITECC: Instituto de la tiroides y Enfermedades de Cabeza y Cuello
ATA: American Thyroid Association
TN: Thyroid nodules
FNA: Fine-needle aspirations
US: Ultrasonographic
IQR: Interquartile range
SD: Standard deviation
TSH: Stimulating thyroid hormone
fT4: Free thyroxine
